# Chronic abdominal vagus stimulation increased brain metabolic connectivity, reduced striatal dopamine transporter and increased mid-brain serotonin transporter in obese miniature pigs

**DOI:** 10.1186/s12967-019-1831-5

**Published:** 2019-03-12

**Authors:** Charles-Henri Malbert, Mickael Genissel, Jean-Louis Divoux, Christine Henry

**Affiliations:** 1 Aniscan Unit, Dept of Human Nutrition, INRA, 35590 Saint-Gilles, France; 2 Pegase Unit, Dept of Animal Physiology, INRA, Saint-Gilles, France; 3 Axonic, Vallauris, France; 4 Livanova, Sorin CRM, Clamart, France

**Keywords:** Vagal stimulation, Bariatric surgery, SERT, DAT, Connectivity analysis, PET imaging, SPECT imaging, Animal model

## Abstract

**Background/objective:**

Changes in brain metabolism has been investigated thoroughly during unilateral cervical chronic vagal stimulation in epileptic or depressive patients. Bilateral stimulation of the abdominal vagus (_a_VNS) has received less attention despite the reduction in body weight and an altered feeding behavior in obese animals that could be clinically relevant in obese individuals. Our study aims to examine the changes in brain glucose metabolism (CMR_glu_) induced by _a_VNS in obese adult miniature pigs. Dopamine (DAT) and serotonin transporters (SERT) were also quantified to further understand the molecular origins of the alterations in brain metabolism.

**Subjects/methods:**

Pairs of stimulating electrodes were implanted during laparoscopy on both abdominal vagal trunks in 20 obese adult’s miniature pigs. Half of the animals were permanently stimulated while the remaining were sham stimulated. Two months after the onset of stimulation, dynamic _18_FDG PET and ^123^I-ioflupane SPECT were performed. Food intake, resting energy expenditure and fat deposition were also assessed longitudinally.

**Results:**

Food intake was halved and resting energy expenditure was increased by 60% in _a_VNS group compared to sham. The gain in body weight was also 38% less in _a_VNS group compared to sham. Brain metabolic connectivity increased between numerous structures including striatum, mid-brain, amygdala and hippocampus. On the contrary, increased CMR_glu_ were restricted to the thalamus, the periaqueducal grey and the amygdala. DAT binding potential was decreased by about one third in the striatum while SERT was about doubled in the midbrain.

**Conclusions:**

Our findings demonstrated that _a_VNS reduced weight gain as a consequence of diminished daily food intake and increased resting energy expenditure. These changes were associated with enhanced connectivity between several brain areas. A lower striatal DAT together with a doubled mid-brain SERT were likely causative for these changes.

**Electronic supplementary material:**

The online version of this article (10.1186/s12967-019-1831-5) contains supplementary material, which is available to authorized users.

## Background

Obesity and its consequences are a worldwide public health issue especially because effective reversible bariatric procedures or pharmacological treatments without serious adverse-effects are still in infancy [1]. Central neuromodulation using vagal stimulation might become an alternate therapeutic tool [2] since it increased satiation [3], reduced food intake [2] and increased energy expenditure [4] without adverse side effects [5]. Furthermore, it restored insulin sensitivity in obese animals and improved the insulin-dependent glucose metabolism of several organs including the brain [6]. The maximum effectiveness towards weight loss was observed with bilateral stimulation performed at the level of the abdominal branches (_a_VNS) [7]. Therefore, it is significantly more complicated than the one used for refractory epilepsy or depression which involve stimulation of the left cervical vagus only [8]. However, since it was performed below the cardiac branches, it avoids unwanted cardiac side effects [5].

While the impact of _a_VNS on brain function is largely unknown, several studies have investigated the effect of left cervical VNS on brain function using PET and fMRI imaging both in animals and humans. In epileptic or depressive patients, cervical VNS induced a gradual brain response involving a change in dorsolateral prefrontal/cingulate cortical activities followed by dopaminergic activation of the limbic system [9] or limbic-connected structures [10, 11]. A widespread impact involving several functional brain networks [12, 13] has been also identified. Opposite to those results, the effects observed in obese animals, during insulin stimulation, after long-lasting _a_VNS were restricted to fewer brain areas involving the amygdala, the dorsal cingulate cortex and the anterior prefrontal cortex [6]. Unfortunately, the different experimental conditions make challenging to ascertain for significant differences of brain response/metabolism during abdominal vs. cervical VNS.

The mesolimbic dopamine reward system plays a central role in the regulation of eating behavior [14] and dopamine receptors availability was lower in morbidly obese individuals [15]. Conversely, cervical VNS increased extracellular striatal dopamine levels [9, 16]. Therefore, striatal dopamine changes induced by VNS could be pivotal to explain the reduced food intake observed during _a_VNS. Furthermore, in addition to dopamine, the other brain neurotransmission system—serotonin might be altered by vagal stimulation. Serotonin transporter (SERT) activity in the dorsal raphe nucleus of the dorsal mid-brain has been identified recently as a critical player for the control of food intake [17]. Former studies have also demonstrated that the activity of SERT within the mid-brain was altered by cervical unilateral vagal stimulation [18]. Taken together, these data suggest that mid-brain SERT in conjunction with striatal DAT might be responsible for the VNS_a_ reduced food intake and body weight.

Our study aimed to characterize the effects of prolonged bilateral stimulation of the abdominal branches of the vagus on brain activity in a large animal model of acquired obesity. Since vagal stimulation is likely to alter the metabolism of specific brain areas as well as the organization of several brain networks [12, 19], the analysis of PET ^18^FDG images will be performed using both statistical parametric and network mapping. Furthermore, the binding potential for dopamine (DAT) and serotonin (SERT) transporters was investigated using quantitative dynamic SPECT imaging after the administration of ^123^I-Ioflupane. We perform our experiment on Yucatan mini-pigs, a model known to develop obesity in response to high fat/sucrose diet [20], that is bulky enough to allow placement of stimulating electrodes around the abdominal vagal trunks using laparoscopy [7] and with relatively large brain with well-defined cerebral gyri [21, 22].

## Materials and methods

The experiment was conducted in accordance with the current ethical standards of the European legislation after approval by the Ethics Committee of Rennes (R-2011-MO-01).

### a. Experimental design and animal model

A total of 20 Yucatan mini-pigs, aged 3 years, were used throughout the experiment. Several studies suggested that DAT availability was higher in females than in males [23]. Therefore, we decided to use only female animals in our experiment. The animals were made obese using high fat, high sucrose diet (4024 kcal/kg of feed) supplied at 150% of the recommended caloric intake (288 kcal/kg BW^0.75^) [24]. This feeding scheme was maintained during the entire experiment.

Once obese, i.e., after 5 months of the obesogenic diet, the animals were fitted by laparoscopy with stimulating electrodes around the abdominal vagal trunks connected to a subcutaneous stimulator (see “b. Vagal stimulation”). Ten animals, selected at random, were vagally stimulated while a nonfunctional stimulator was inserted in the remaining ten animals. The stimulation was started immediately after surgery and continued for 2 months. During this period, the food intake and the electrodes/stimulator impedance were recorded daily. Body weight was measured every week. Then ^18^FDG PET and ^123^I-ioflupane SPECT imaging were performed at least 4 days apart. Additional phenotypic characterization of the obesity in both groups was achieved during terminal anesthesia.

### b. Vagal stimulation

Two sets of cuff electrodes were fixed on the dorsal and ventral vagal trunks using laparoscopy as previously described [7]. The stimulating electrodes consisted of in-cuff electrodes for a nerve diameter target of about 3.0 mm and comprised two pairs of Pt–Ir 10% half circular contacts (4 in total), short-circuited together to form a bipolar configuration. Pairs of contacts were located on both sides of a tube, creating a circumference, 10 mm distal from the other pair of contacts. The overall dimension of the cuff was 25 ± 0.1 mm.

The electrode leads were connected to a neurostimulator that was implanted in a subcutaneous pocket behind the last left rib. The neurostimulator was able to deliver a current-controlled pulse to the two set of electrodes. It included an impedance measuring subroutine that allowed the maximum and minimum impedance values to be recorded daily. Stimulation was delivered as biphasic squared pulse trains. The frequency of the pulses within the train was 30 Hz for a 500 ms pulse. The duration of each pulse train was 30 s, and the interval between pulse trains was 5 min. The amplitude of the pulse was initially set to 1 mA, after surgery to progressively reach 5 mA 1 week later. This value was maintained until the end of the experiment.

### c. ^18^FDG image acquisition and analysis

Brain metabolism was quantified using PET imaging after ^18^Fluoro-deoxyglucose injection. PET images were acquired on an ECAT EXACT HR CTI/Siemens scanner while the animal was anesthetized using isoflurane according to a previously published procedure [6]. Axial projections were corrected for dead time, decay and photon attenuation. Cerebral metabolic rate for glucose utilization (CMRglu) coded images were obtained using the dynamic PET images series and the arterial input function using Pmod 3.8 (PMod, Switzerland) adapted to the porcine brain. A value of 0.45 was selected for lump constant of the porcine brain [25] and 1.05 g/ml for the density of the brain tissue. Glycemia during the PET imaging was obtained from the arterial samples and measured using the glucose oxidase method on a rapid analyzer (Analox GM9 Analyzer; Analox Instruments, London, UK). The CMRglu coded image set obtained using FDG compartment modeling was used afterwards for the quantitative analyses.

Statistical analyses of glucose brain uptake images were performed using SPM12 with significance level set at P < 0.001 FDR corrected to exclude random brain activation [26]. Brain templates used for registration procedure were those described earlier [6] and were within the same tridimensional reference as our pig brain atlas [22]. Metabolic connectivity analysis was also performed using the CMRglu coded image set. Network components were obtained using NetPET V1 (L. Moro, M. Veronese and M. Arcolin) according to Rubinov and Sporns [27, 28]. Brain networks (nodes and edges) were visualized using BrainNet Viewer [29] and the circularGraph Matlab routine. Nodes locations were obtained from the barycenter of volumes of interest describing the major brain structures obtained using the pig brain atlas (see Additional file 1: Table S1). Weight based thresholding for edges was used for statistical analysis together with the corresponding probability matrix [30].

### d. ^123^I-Ioflupane image acquisition and analysis

Dopamine and serotonin binding potential was quantified using dynamic SPECT imaging after ^123^I-ioflupane injection (180 MBq, DatScan, GE). Brain SPECT was achieved with a double head gamma camera (Millenium VG, GE) in isoflurane-anesthetized animals. Immediately after the intravenous administration of ^123^I-ioflupane, 180-degree acquisitions were performed (3° Step, 45 s per frame, 128*128 pixels per frame) sequentially during about 6 h resulting in eight projection series used afterwards to reconstruct a dynamic (4D) brain sequence. The corresponding projections were reconstructed using OSEM algorithm with NiftyRec/Matlab software and filtered afterward with a non-local means filter. Co-registered CT scan brain images (HiSpeed CT, GE) transformed as attenuation maps for the ^123^Iodine photopeak were integrated into the NiftyRec projector to remove attenuation. Scatter compensation was also achieved using the Jaszczak subtraction method [31].

Dopamine transporter and SERT binding potentials (unitless) were obtained from 4D SPECT images using Ichise Multi-linear Reference Tissue Model [32] implemented in PXMOD software with reference region being the cerebellum crus [33]. DAT and SERT areas were defined from the volumes of interest extracted using the pig brain atlas. The DAT rich areas embraced the Putamen and the Caudate nucleus whereas the SERT rich areas included the Pons and the MidBrain. Left and right structures were merged into a single volume for DAT and SERT rich volume of interest (VOI). The rationale for the classification of the volumes into DAT and SERT rich areas followed the work of Peremans [33] and Roselli [34] adapted to the anatomical particularities of the porcine brain. Differences between binding potential in sham and stimulated group were analyzed using T-test with P < 0.05 as threshold for significance.

### e. Ancillary measurements

Phenotypic characterization of the obesity status was performed during the last anesthesia procedure performed also used for imaging. It includes the evaluation of the whole-body metabolism by indirect calorimetry and the analysis of fat tissue mass using CT scan. Energy expenditure and carbohydrate oxidation rate were measured by indirect calorimetry. A breath to breath metabolic analyzer (Quark RMR Cosmed) was attached to a non-rebreathing ventilator (Siemens SAL 900) to measure the difference between inspired and expired VO2 and VCO2. The energy expenditure and carbohydrate oxidation rate were determined, as described previously [6]. Fat mass repartition and lean mass were quantified by semi-automatic segmentation (Osirix 8.0) of CT-based images of the abdomen obtained at the levels of L1 and L3 (HighSpeed CT, GE) [35].

### f. Non-image statistical analyses

Non-image data are presented as mean ± SE and compared by T-tests using Prism 7 (GraphPad, USA). Time-dependent analysis were corrected for multiple comparisons using the Sidak test. Differences were regarded as statistically significant if P < 0.05.

## Results

Adverse events associated either to the implantation or the stimulation were not observed during the course of the experiment.

### a. Effect of abdominal bilateral VNS on body weight and metabolic balance

Electrode impedance was 834 ± 130 Ohms immediately after implantation and steadily increased up to 1185 ± 182 Ohms during the first 4 weeks after surgery. There was no difference between the impedances recorded on the dorsal vs ventral electrodes. Similarly, the changes in impedance were not significantly different between the sham and the stimulated group (P < 0.05).

**Fig. 1:**
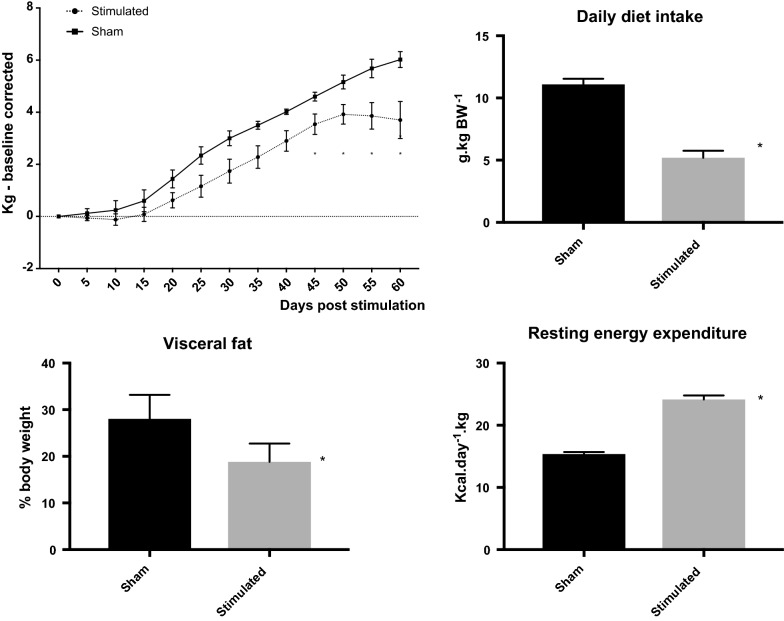
Evolution in body weight gain for sham and abdominal vagal stimulated groups (top left panel). Note that body weight relative to the pre-operative one was significantly reduced in the stimulated group after 45 days of continuous stimulation. This reduction was a consequence of both a diminished daily food intake (top right panel) and an increased resting energy expenditure (bottom right panel). The decreased body weight in the stimulated group was representative of a reduced visceral fat (bottom left panel)

All animals gained weight after surgery irrespective of the experimental group but it was less for stimulated than for sham group. The difference was significant 45 days after surgery (3.5 ± 0.39 kg vs. 4.6 ± 0.17 kg, P < 0.01 for stimulation vs. sham group) and continue to be so until the end of the experiment (3.7 ± 0.71 kg vs. 6.0 ± 0.30 kg, P < 0.01 for stimulation vs. sham group at 60 days). Diet intake, averaged for the experimental period, was about halved in stimulated versus sham group (473 ± 24.6 g/day vs. 810 ± 22.5 g/day, P < 0.01). Together with an increased in resting energy expenditure (2120 ± 127.4 kcal/day vs. 1305 ± 103.0 kcal/day, P < 0.01) observed in stimulated versus sham group, the daily energy deficit was 216 ± 21.3 kcal in the stimulated group. Fat deposit was decreased in stimulated group as a consequence of a decreased in visceral fat (Fig. 1). Subcutaneous fat was unchanged between groups (P > 0.05).

### b. Changes in brain glucose metabolism

**Fig. 2:**
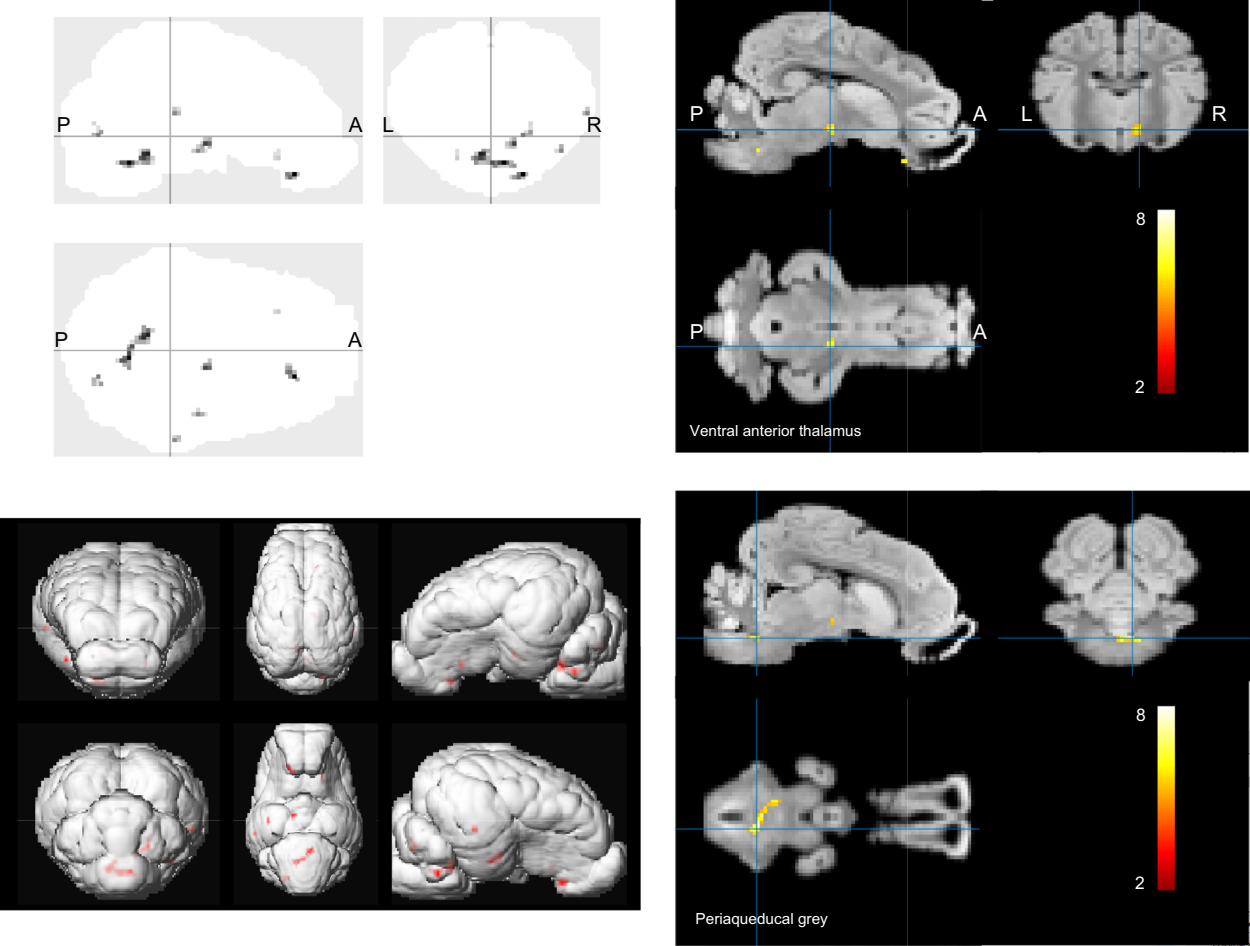
Statistical parameter mapping of ^18^FDG uptake (CMRglu) in sham and stimulated group. SPM analysis was performed using pixel-wise modeled quantitative PET images. Statistical differences were analyzed using T-test in SPM12, P < 0.001 FDR and cluster level corrected. The analysis was performed with a cluster size of 100 voxels each of the cluster representing 1 mm^3^. Note the activation in the thalamic and periaqueducal grey areas. Top left—brain glass with activation in shade of grey. Top right—thresholded activation (in color scale) depicting the ventral anterior thalamic activation. Bottom left—3D representation of the activation matrix. Shade of red indicates activation. Bottom right—thresholded activation (in color scale) with the image centered on the periaqueducal grey

Statistical parameter mapping performed on quantitative CMRglu images showed that brain glucose uptake was increased in the stimulated group but for a limited number of brain areas only. They included the periaqueducal grey, the thalamic and hypothalamic areas and part of the amygdala and the insular cortex (Table 1, Fig. 2). Increasing P values at 0.01 resulted in extended activation of the periaqueducal grey and additional activations of the inferior temporal gyrus, the prepyriform area and the striate cortex (T = 3.307).

**Table 1:** Statistical parameter mapping analysis of activation patterns (local maxima) obtained from ^18^FDG PET scan in stimulated animals compared to sham

Coordinate of local maximum (x, y, z in mm)	P_corrected_ (voxel level)	T-value (voxel level)	Tentative anatomic localization
2, − 11, − 8	0.001	7.46	Periaqueducal grey
− 4, − 6, − 6	0.001	6.96	Periaqueducal grey
6, 9, − 2	0.001	6.32	Ventral anterior thalamus R
− 0, 9, − 9	0.003	5.46	Medial hypothalamic area
− 18, 7, − 4	0.002	6.06	Amygdala L
− 10, 28, − 6	0.004	4.88	Insula L

The analysis was performed with the cluster size of 100 voxels. Values of P were presented using FDR correction. Tentative anatomic localization is given based on interpretation of the projection of the activation pattern on the pig brain anatomic atlas published by our group [22]

**Fig. 3:**
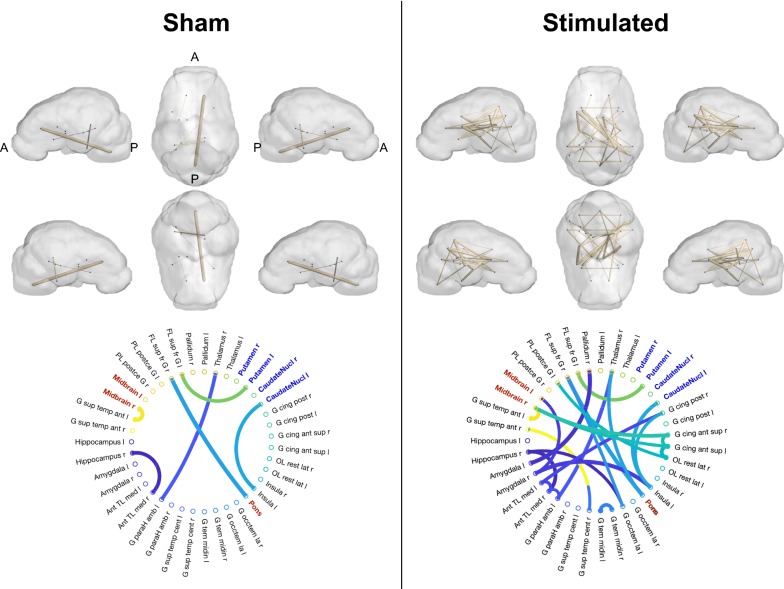
Metabolic connectivity analysis in the sham and stimulated groups. A. The figures showed the results of connectivity analysis performed with NetPET software using CMRglu coded voxel-vised images. Top panels—surface renderings of the brain overlaid with network connections. Nodes and edges were coded using BrainNet viewer and edges were thresholded for P < 0.001. Only nodes with attached edges were represented. Bottom panels—Correlograms representing the correlation weighted matrices thresholded at P < 0.001. Nodes’ abbreviations are expended in the Additional file 1: Table S1. Note the increased metabolic connectivity in stimulated compared to sham group

Correlation analysis showed that metabolic connectivity was significantly enhanced in stimulated versus sham group as indicated by significantly different matrices of pair-wise correlations. Several networks were identified in the stimulated group (Fig. 3). The connectivity with the hippocampus and the amygdala formed a strong functional network with the anterior thalamic area. Similarly, we identified a significant connectivity between the cingulate structures, the postcentral gyrus, the insular cortex and the mid-brain. There was little connectivity involving the striatum either in the sham or in the stimulated group.

### c. Changes in DAT and SERT binding potential

**Fig. 4:**
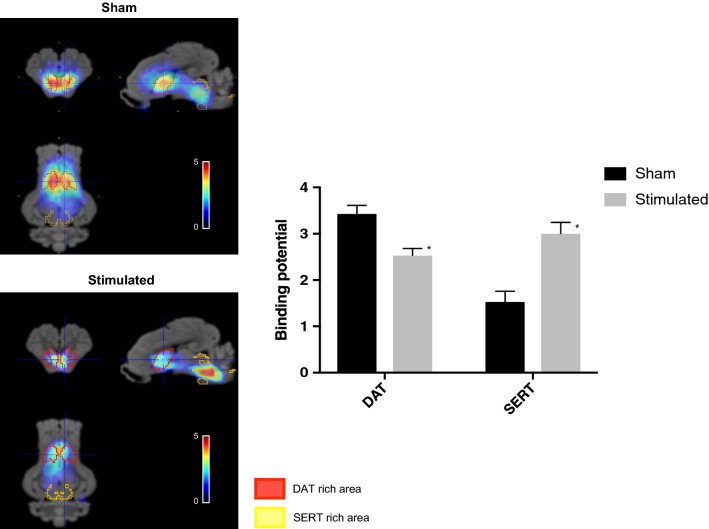
Left panels—examples of reconstructed normalized volumes obtained after the administration of ^123^I ioflupane in sham versus stimulated animals. The colors represented pixel-wise modeled SPECT dynamic image series according to Ichise i.e., the binding potential overlaid on the MRI template of the pig brain atlas. Red VOIs corresponded to DAT-rich areas whereas yellow VOIs represented SERT-rich areas. Right panel—binding potential for DAT and SERT in sham versus stimulated groups obtained from region-based analysis. *Indicates a significant difference at P < 0.001

The binding potential of DAT (in DAT-rich areas) was significantly decreased in stimulated versus sham group whereas that of SERT (in SERT-rich areas) increased (Fig. 4). DAT binding decreased by 26% whereas SERT binding about doubled in stimulated group compared to the sham. There was very little binding (lower than 0.2) outside DAT and SERT rich areas and this was not different between groups (P = 0.120).

## Discussion

Our study demonstrates that bilateral abdominal vagal stimulation generated a unique brain response associated with reduced body-weight gain, diminished daily food intake, lowered visceral fat and increased resting energy expenditure in obese non-diabetic animals. This central response is characterized by an extensive increase in brain connectivity whereas statistical parameter mapping identified only limited changes in CMRglu. The molecular mechanism responsible for these patterns involved a doubling in SERT binding at the Mid-brain/Pons whereas DAT binding within the striatum was significantly reduced.

Bilateral vagal stimulation performed at the abdominal level reduced body-weight gain in obese animals. This was confirmed in experimental conditions limiting false positive i.e., (i) large number of animals, (ii) extended duration of stimulation, (iii) procedure that avoided tissue damage and improved surgical recovery. The weight reduction was quantitatively small and occured several weeks only after the onset of continuous stimulation. However, compared to marketed drug therapies for chronic weight management, vagal stimulation was performing similarly than the best pharmacotherapy available [36]. Indeed, the marketed drugs achieved 3% to 9% weight loss, relative to placebo, after 1 year. This weight loss translates into about 32 g per day while in our experiment, the animals loosed 38.7 g per day compared to sham. Furthermore, the energy deficit observed in stimulated animals was within the range of the required energy deficit per unit weight loss in patients. Indeed, one of the most pervasive weight loss rules states that a cumulative energy deficit of 3500 kcal is necessary to lose 1 lb of body weight [37]. Here, at 60 days post stimulation, the animals have a daily energy deficit of 216 kcal, and the cumulative energy deficit suggests that one kg will be lost in 36 days. These crude calculations did not allow for the ratio between lean and fat mass [38] that acted as an adjustment variable for the equivalence between weight loss and energy deficit. Nevertheless, the convergence of these figures suggests that both feeding behavior and energy expenditure are keys components to understand the origin of weight loss observed during vagal stimulation.

Numerous studies have investigated the effect of unilateral cervical VNS either in healthy animals or in epileptic and depressive patients [39]. While several confounding factors exist in humans’ studies including the analytic methods and the outcome of the disease, the involvement of thalamic, limbic and prefrontal structures were the most consistent findings [40, 41] especially using fMRI imaging. These activations differed from the c-fos or ∆FosB data obtained in healthy rats [42] for which, aside from bulbar activation, frontal, cingulate and amygdala cortices together with part of the striatal system were activated during chronic application of VNS. Surprisingly, bulbar activation, the main entrance for vagal afferents, was only recently identified using noninvasive imaging either in animals with pulson type vagal stimulation [7] or in humans with transcutaneous VNS [43]. Sometimes, the reported activity highlight activations in different regions or even opposite responses in the same regions [44]. It is extremely likely that most of these differences were related to the non-quantitative analysis [45]. The methodological challenge for truly quantitative PET imaging explains probably the oversimplification in imaging procedures. Here, using quantitative CMRglu measurement, we found that most regions activated in abdominal VNS group were located within the thalamic area, the periaqueducal grey and to a lesser extend within the amygdala and the insular cortex. Therefore, our data demonstrated a robust activation of these structures unlike the mixture of activations and deactivations on frontal, limbic, thalamic and mid-brain structures reported during unilateral cervical vagal stimulation.

Network analysis on quantitative CMRglu images identified an increase in the metabolic connectivity for several brain structures well outside those identified by SPM analysis. This change was reminiscent that observed by. Cao et al. [12] who found that acute left cervical VNS reorganized the functional connectivity of the limbic system. However, unlike Cao et al. [12] brain-wide responses were not identified with chronic _a_VNS. The involvement of the amygdala observed both in connectivity analysis and SPM measurements deserves some attention since it has not been identified earlier with _a_VNS. Memory storage processes and neuronal plasticity in the hippocampus have been already observed in rat during vagal stimulation at the cervical level [46]. Furthermore, word-recognition memory was improved by chronic cervical vagal stimulation in humans [47]. All these agreed with a vagally induced increased metabolism of these brain region (e.g. increased CMRglu).

Serotonin transporter binding potential doubled in the mid-brain and pontine areas for the stimulated group. An increased release of serotonin has been already demonstrated in some areas of the mesencephalon during cervical VNS [48]. Since, there is a direct relationship between the synaptic release of serotonin and SERT expression in rats [49], it is likely that the improvement of SERT binding potential reflects an increase of 5HT release as a consequence to _a_VNS. To what extent this phenomenon is beneficial for a reduction in food intake or is the consequence of the reduced body weight is debatable. An inverse relationship between brain serotonin, food intake and body weight has been strongly established [50, 51]. Furthermore, a causal link between the central nervous system biogenic amine targets (namely 5-HT) and the energy expenditure has been established preclinically and clinically [52]. Therefore, it is logical that the increased binding potential of SERT observed in stimulated animals participates in the reduction in body weight together with the increased energy expenditure. The decreased SERT availability in the midbrain of obese women [53] and vice versa the increase of SERT availability following the successful treatment of eating disorder in a subgroup of the same patients [54] confirm this hypothesis. Finally, the recent identification of a mid-brain circuit associated with serotonin release that control food intake in the rat [17] fits adequately within the overall picture of a mid-brain SERT mediated effect of _a_VNS.

Dopamine transporter binding potential was also decreased by one third in the striatum of _a_VNS animals. Unlike SERT, this reduced DAT binding potential translates probably into an enhanced dopamine concentration within the same brain structures dopamine levels increased within the same structures in DAT knockout animals [51, 55]. Unsurprisingly, cervical VNS enhanced extracellular dopamine level in the prefrontal cortex and the striatum of healthy rats [48]. Furthermore, since pharmacologically increased mesolimbic dopamine activity obtained by selective inhibition of DAT was sufficient to suppress feeding [56], it is likely that the decrease DAT binding potential observed in the stimulated group was primarily causative for the reduction in food intake. Nevertheless, the negative correlation between body mass index and DAT1 gene expression that translated into inhibition of DAT binding in obese is somewhat contradictory with the previous observations. However, an absence of correlation was also reported by several authors who investigated exclusively non-diabetic (healthy) obese [57, 58].

Our study has several limitations due to the particularities of the porcine experimental model. First, the obese insulino-dependent pig did not exhibit diabetes unlike other experimental animals while fed with a high-fat high energy diet [59, 60]. Since it is believed that at least half of obese individuals were metabolically healthy, i.e., nondiabetic [61], this unique feature of the porcine model is somewhat positive as it mimics the human pathology. However, because in the obese individual, striatal DAT likely depends on the diabetic vs. nondiabetic status [58, 62], we cannot ascertain that the effect observed on DAT in our model is representative of the human pathological condition. Second, Minuzzi et al. found that two others propane derivative labeled with ^11^C have low pharmacological specificity for DAT in pigs [63]. It is questionable whether this also applies to ^123^I ioflupane but this might have some consequences on the distribution of SERT in SERT-rich areas. Nevertheless, unlike Minuzzi et al. [63], we used several methods that taken together produced quantitative DAT/SERT binding potential measurements: attenuation and diffusion corrections, Ichise multi-linear reference tissue reconstruction with 4D data using extensive acquisition duration. Finally, using the aforementioned techniques, ^123^I ioflupane radioactivity concentrates in the striatum and in the mid-brain/Pons. The amount of binding outside these structures is neglectable in line with the observations performed in other species and in humans [33].

Our study did not investigate different stimulation schemes because, despite their potential interest, dose–response curves were almost impossible to build in our model. Indeed, the impact of VNS towards food intake took several weeks to be significant [3]. Recently, we were able to observe a significant alteration of the feeding behavior after 2 weeks but this was achieved on young animals with a complex stimulation scheme involving high frequency chopped current pulses [7]. Furthermore, no experimental data were available to evaluate an adequate washout period (if any) before changing one stimulation parameter. Finally, due to the intricacy of the numerous parameters constituting a stimulation scheme (pulse duration, pulse frequency, duration of the burst, interval between burst and stimulation current), evaluation of each component individually proved to be impracticable [5].

## Conclusions

Abdominal bilateral chronic vagal stimulation, performed in conditions close to a future clinical setting (i.e., laparoscopic vagal access, stimulation with implanted stimulator lasting several weeks), was able to effectively reduce food intake and body weight. This was associated with brain metabolism changes that differed significantly from those observed during cervical unilateral vagal stimulation. We identified an enhanced brain connectivity involving several areas such as the striatum, cingulate, insula, thalamus, amygdala, hippocampus and mid-brain. These changes occurred together with profound alterations in DAT and SERT binding potentials. DAT binding potential was decreased in the striatum while SERT binding potential was doubled in the mid-brain. All of the above might be pivotal to explain the origin of the reduced food intake and diminished weight gain induced by _a_VNS. Furthermore, notwithstanding the application of _a_VNS for morbid obesity, these changes gave new avenues for drug-based therapies targeted towards control of food intake in the obese.

## Additional file

**Additional file 1: Table S1.** Abbreviation list of the 39 regions used in the VOI evaluation based on the Saikali et al. (1) pig atlas. The order and definition of the regions were extracted from Hammers et al. (2) human brain atlas and were tentatively applied to the pig atlas.

## Abbreviations

aVNS: chronic bilateral abdominal vagal stimulation
FDR: false detection rate
CMRglu: cerebral metabolic rate for glucose utilization
DAT: dopamine transporter
SERT: serotonin transporter
