# Supplementary material for: Chronic abdominal vagus stimulation increased brain metabolic connectivity, reduced striatal dopamine transporter and increased mid-brain serotonin transporter in obese miniature pigs
Source: J Transl Med. 2019 Mar 12;17:78. doi: 10.1186/s12967-019-1831-5 (PMC6417219; doi:10.1186/s12967-019-1831-5)
Supplement: Supplementary file 1 — Additional file 1: Table S1. Abbreviation list of the 39 regions used in the VOI evaluation based on the Saikali et al. (1) pig atlas. The order and definition of the regions were extracted from Hammers et al. (2) human brain atlas and were tentatively applied to the pig atlas. [file 12967_2019_1831_MOESM1_ESM.docx]

**Additional file**

Table S1. *Abbreviation list of the 39 regions used in the VOI evaluation based on the Saikali et al* (1) *pig atlas. The order and definition of the regions were extracted from Hammers et al* (2) *human brain atlas and were tentatively applied to the pig atlas.*

| **Abbreviation** | **Complete Name** |
| --- | --- |
| Hippocampus l | Hippocampus (left) |
| Hippocampus r | Hippocampus (right) |
| Amygdala l | Amygdala (left) |
| Amygdala r | Amygdala (right) |
| Ant TL med l | Anterior temporal lobe, medial part (left) |
| Ant TL med r | Anterior temporal lobe, medial part (right) |
| G paraH amb l | Parahippocampal and ambient gyri (left) |
| G paraH amb r | Parahippocampal and ambient gyri (right) |
| G sup temp cent l | Superior temporal gyrus, central part (left) |
| G sup temp cent r | Superior temporal gyrus, central part (right) |
| G tem midin l | Middle and inferior temporal gyrus (left) |
| G tem midin r | Middle and inferior temporal gyrus (right) |
| G occtem la l | Fusiform (lateral occipitotemporal) gyrus (left) |
| G occtem la r | Fusiform (lateral occipitotemporal) gyrus (right) |
| Pons | Pons |
| Insula l | Insula (left) |
| Insula r | Insula (right) |
| OL rest lat l | Lateral remainder of occipital lobe (left) |
| OL rest lat r | Lateral remainder of occipital lobe (right) |
| G cing ant sup l | Cingulate gyrus, anterior part (left) |
| G cing ant sup r | Cingulate gyrus, anterior part (right) |
| G cing post l | Gyrus cinguli, posterior part (left) |
| G cing post r | Gyrus cinguli, posterior part (right) |
| CaudateNucl l | Caudate nucleus (left) |
| CaudateNucl r | Caudate nucleus (right) |
| Putamen l | Putamen (left) |
| Putamen r | Putamen (right) |
| Thalamus l | Thalamus (left) |
| Thalamus r | Thalamus (right) |
| Pallidum l | Pallidum (left) |
| Pallidum r | Pallidum (right) |
| FL sup fr G l | Superior frontal gyrus (left) |
| FL sup fr G r | Superior frontal gyrus (right) |
| PL postce G l | Postcentral gyrus (left) |
| PL postce G r | Postcentral gyrus (right) |
| MidBrain l | MidBrain (left) |
| MidBrain r | MidBrain (right) |
| G sup temp ant l | Superior temporal gyrus, anterior part (left) |
| G sup temp ant r | Superior temporal gyrus, anterior part (right) |

1. Saikali S, Meurice P, Sauleau P et al. A three-dimensional digital segmented and deformable brain atlas of the domestic pig. J Neurosci Methods. 2010;192:102-109.

2. Hammers A, Allom R, Koepp MJ et al. Three-dimensional maximum probability atlas of the human brain, with particular reference to the temporal lobe. Hum Brain Mapp. 2003;19:224-247.
